# Surgical Outcome of Distally Based Peroneus Brevis Flap: A Retrospective Study

**DOI:** 10.7759/cureus.26329

**Published:** 2022-06-25

**Authors:** Joyal Jose, Sabu C Parameswaran, Aniraj Rajappan, Lisha N Prameela

**Affiliations:** 1 Department of Plastic and Reconstructive Surgery, Government Medical College, Thiruvananthapuram, IND

**Keywords:** lower-limb reconstruction, lower leg defects, muscle flap, distally based flap, peroneus brevis flap

## Abstract

Background: This study aimed to evaluate the surgical outcomes of distally based peroneus brevis muscle flap in post-traumatic lower leg defects.

Methods: This is a retrospective analysis conducted from February 2017 to May 2019 including six patients who had sustained post-traumatic lower leg critical soft tissue defects and were treated with distally based peroneus brevis muscle flap and primary skin grafting.

Results: In five cases, the flap was successful in providing excellent soft tissue cover to the defects addressed. Partial flap loss was seen in one patient. Skin graft partial loss was seen in three of the six patients. There was no significant donor site morbidity.

Conclusions: The distally based peroneus brevis muscle flap is a safe, easy, and reliable flap option for coverage of lower leg defects. The muscle flap also thins with time to provide a good aesthetic outcome.

## Introduction

Skin and soft tissue defects in lower one-third of leg and ankle have always been a challenge for reconstructive surgeons. Limited number of loco-regional options, scars of previous surgeries, cost of surgery, prolonged surgical time, and lack of adequate vessels for free flap transfer are the concerns in these defects. Though free flaps are commonly employed in lower leg defects, they require an optimized patient, surgical expertise, sacrifice of a major vessel especially when end-to-side anastomosis is not possible, cost implications, prolonged surgical time, etc.

The distally based peroneus brevis flap (DBPBF) is described in literature as a good option for coverage of small to medium-sized defects of lower leg and ankle and also various modifications of the same are described, with an acceptable outcome. This study was intended to present our clinical experience with this flap to evaluate the surgical outcome of distally based peroneus brevis muscle flap in post-traumatic lower leg defects.

## Materials and methods

A retrospective analysis of all cases of DBPBFs done at our center from February 2017 to May 2019 for post-traumatic critical lower leg defects (defects with underlying bare tendon or bone exposure that cannot be managed by skin grafting alone) was done. Patient characteristics, comorbidities, associated injuries, defect location, and size were noted. Arterial Doppler study (to visualize adequate distal perforators to the peroneus brevis flap) and an x-ray of the affected limb were done in all cases. High-velocity injuries with extensive zone of trauma and cases in which an adequate distal perforator was unavailable were excluded from the study. All the cases were done by a single surgeon, with his team. A total of six cases were included, satisfying the above criteria. Intra-operative details were noted and post-operative complications were noted. Follow-up period varied from 27 months to two months.

Surgical anatomy

Peroneus brevis is located in the lateral compartment of leg, deep and anterior to the peroneus longus. Muscle origin is from the middle and lower one-third of the lateral surface of fibula, anterior and posterior inter-muscular septum, and inserts at the styloid process at the base of the fifth metatarsal. Nerve supply is by the superficial peroneal nerve (L5, S1). Peroneus brevis is classified as both type II and type IV muscle in literature [1-3]. This flap was originally thought of having class II vascular pattern but was later reclassified to type IV. Various cadaveric studies also supported this type IV vascular pattern.

Markings and surgical technique

**Figure 1:**
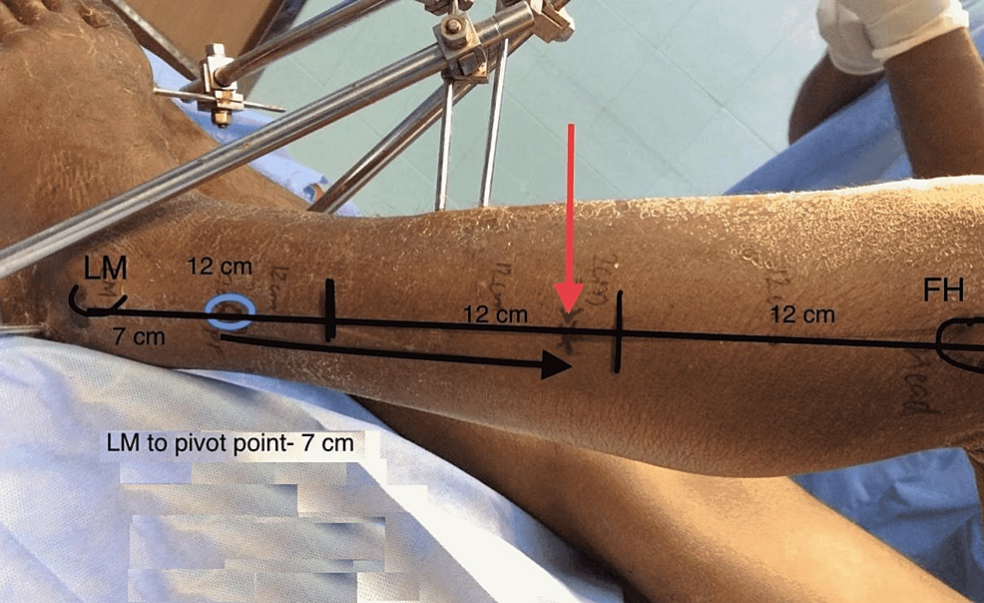
Markings for a distally based peroneus brevis muscle flap (blue circle: pivot point; red arrow: main pedicle; black arrow: flap length) LM: lateral malleolus; FH: fibula head

A straight line is drawn from the tip of lateral malleolus to fibular head and this length is divided into thirds. Proximal vascular supply is approximately located 2 cm below the upper one-third to lower two-thirds junction of this line, which roughly corresponds to the upper limit of the flap, for planning purpose. The pivot point of this flap is located at 6-8 cm from the lateral malleolus along this line, which roughly corresponds to the entry point of the distal most perforator to the peroneus brevis muscle. Flap length approximately corresponds to the distance between pivot point and the point of entry of proximal vascular supply (actual flap length can only be determined intraoperatively, depending on the extent of attachment to the fibula shaft). The markings for the flap are shown in Figure 1.

**Figure 2:**
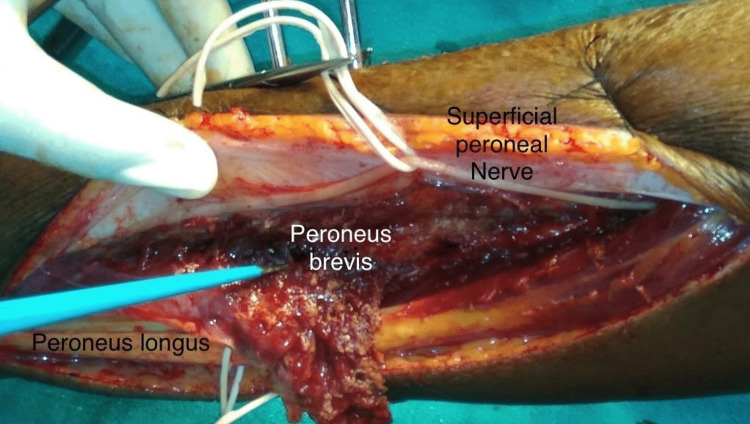
Surgical dissection of peroneus brevis muscle from lateral surface of fibula

Under tourniquet control, a curvilinear incision is made centered 2 cm posterior to the line joining lateral malleolus to fibular head corresponding to the flap length as planned. Skin and subcutaneous tissue are incised with due care taken to avoid damaging the subcutaneous course of superficial peroneal nerve. Deep fascia is incised over the peroneal compartment and subfascial course of superficial peroneal nerve is identified and safeguarded. The first muscle and tendon that we see will be the peroneus longus muscle which is retracted posteriorly to expose the peroneus brevis muscle. The two muscles are separated to visualize the neurovascular pedicle of peroneus brevis. The neurovascular pedicle is isolated, ligated and muscle separated from lateral surface of fibula using bipolar cautery (Figure 2).

The muscle is dissected distally frequently checking the reach into the defect. Once the reach is found to be adequate, the tourniquet is released, hemostasis attained and vascularity of the distal end of the flap (proximal portion of the muscle) confirmed. The vascularity of the distal end may be dubious and might need some trimming, till healthy bleeding is seen. The intervening skin bridge between the pivot point of the flap and the defect can either be incised or the flap can be tunneled to the defect. After tunneling, muscle anchoring to the skin distal to the defect was done for additional flap hold and split thickness skin grafting was done after flap inset. Skin graft donor site chosen was ipsilateral lateral leg. Flap donor site was closed with 3-0 polyglactin sutures for subcutaneous tissues and 4-0 polyamide interrupted mattress sutures for the skin. Skin graft donor site was dressed with paraffin gauze in usual manner.

The grafted skin was secured with sutures or staplers and bulky dressing done, taking care to avoid shearing. The limb was immobilized in the below knee posterior slab. Post-operative limb elevation was done in all cases. Flap assessment was done on post-operative day four, and all sutures were removed by day 14. Flap donor site was managed by scar massage.

## Results

Patient characteristics, comorbidities, size and location of defect, flap/graft loss, donor site morbidity, and any additional procedures required were tabulated (Table 1).

**Table 1:** Patient characteristics, wound characteristics, flap/graft loss, and any additional morbidity/procedures required are summarized DM: diabetes mellitus; HTN: hypertension; SSG: split skin grafting; NPWT: negative pressure wound therapy

S. no.	Age/sex	Comorbidity	Location of defect	Size of defect	Flap loss	Graft loss	Donor site morbidity	Additional procedures
1	78/M	DM, HTN	Right lower 1/3rd tibia-medial aspect	4×3cm	-	Partial	-	SSG
2	45/M	DM	Right lower 1/3rd tibia- Anterior aspect	3×2cm	-	-	-	-
3	16/M	-	Right medial malleolus	4×3cm	At tip (non-critical)	Partial	-	NPWT+SSG
4	42/M	-	Left lower 1/3rd tibia- medial aspect	4×3 cm	-	Partial	-	-
5	60/M	DM	Right lower 1/3rd tibia medial aspect	5×2cm				
6	44/M	-	Left lower 1/3rd tibia anterior aspect	3×2cm	-	-	-	-

There were no cases of critical flap loss. Partial graft loss was seen in three patients, of which two patients required repeat grafting after few weeks. One patient required negative pressure wound therapy (NPWT) before skin grafting, and another patient required dermotaxis for complete wound closure. Case number 4 is shown in Figures 1, 2.

**Figure 3:**
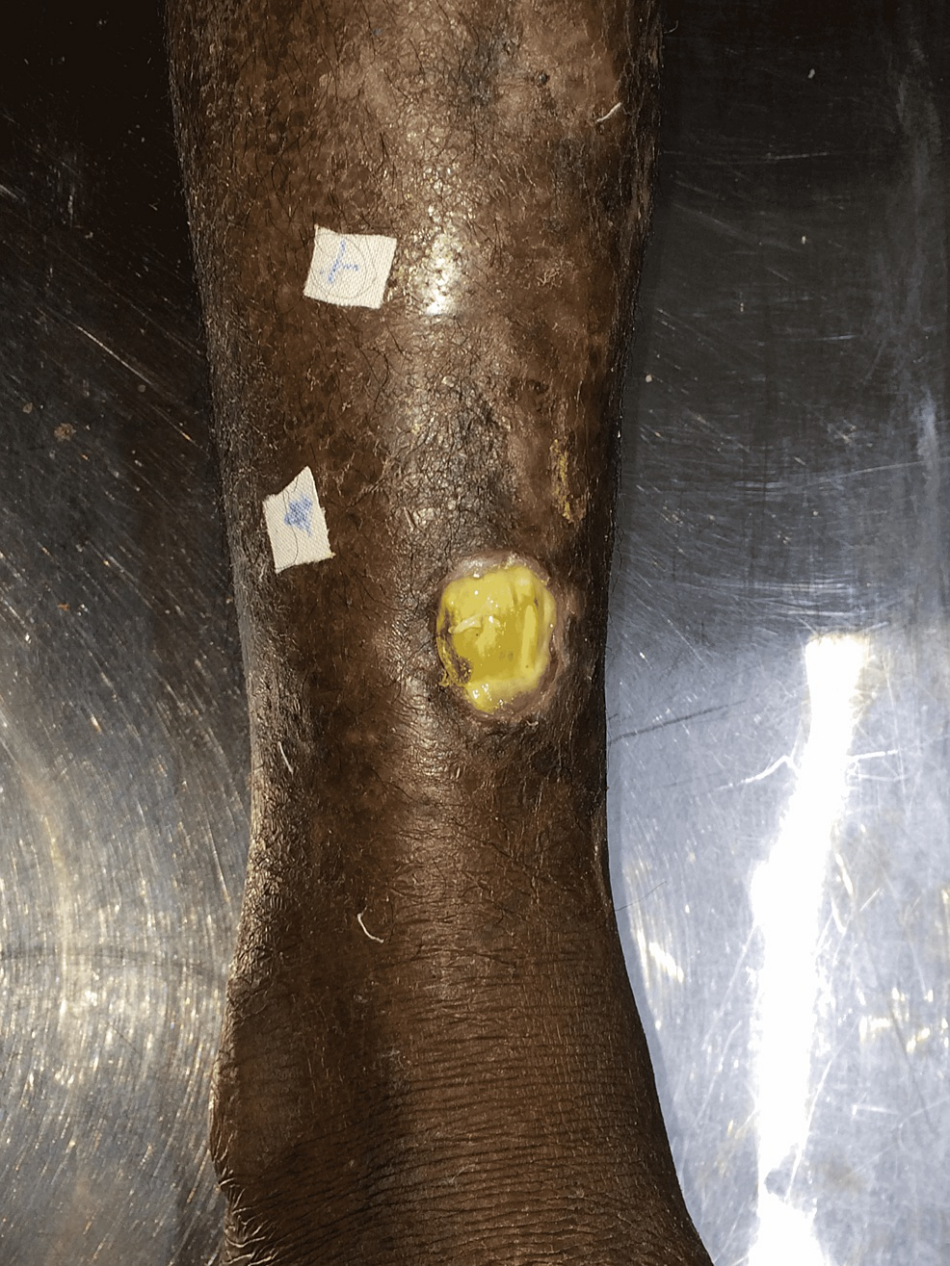
Pre-operative defect of case 2 demonstrating a 3×2 cm defect of lower one-third leg anterior aspect, with underlying tendon exposure

**Figure 4:**
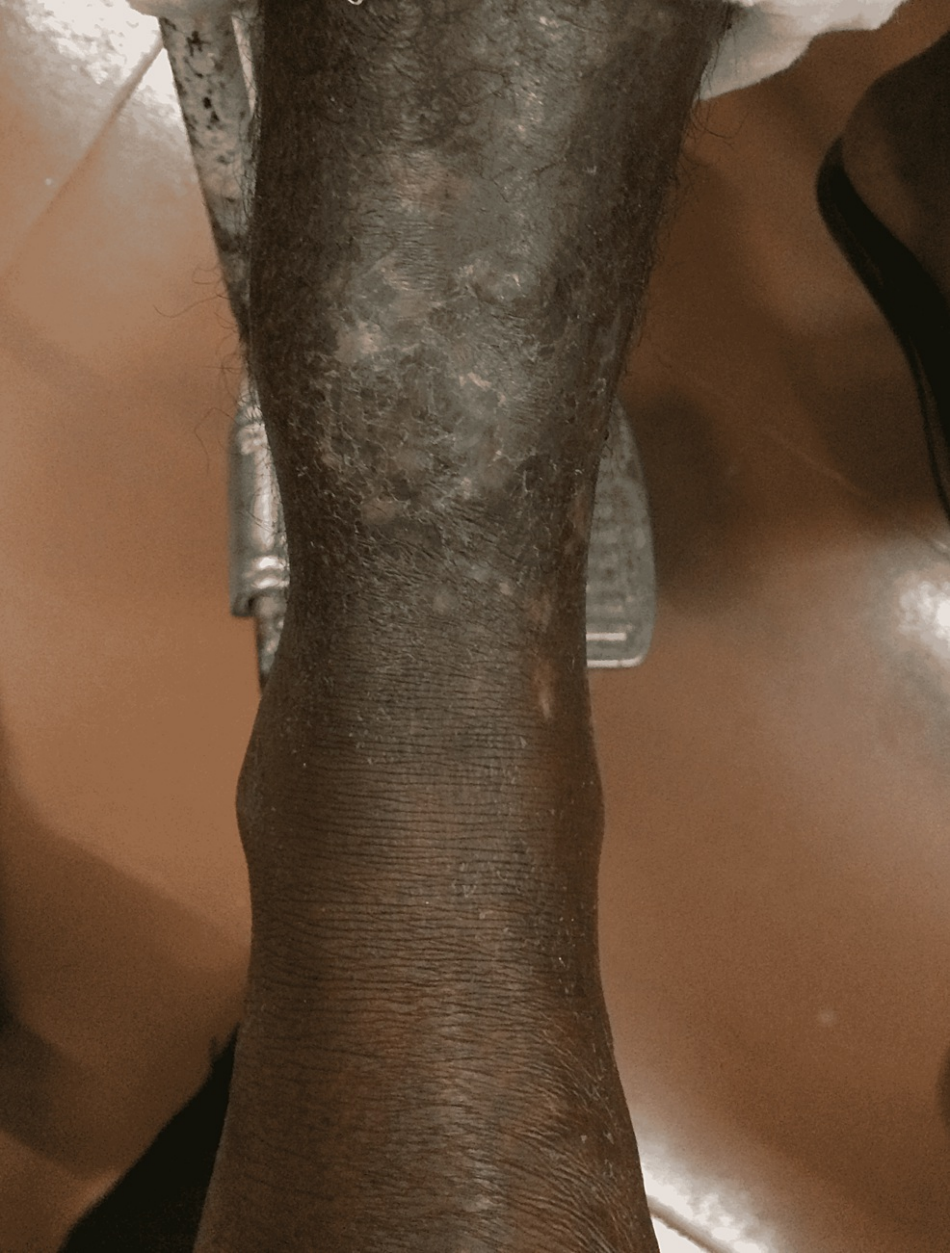
Six months post-operative appearance of case 2 demonstrating auto-thinning phenomenon of the flap

Pre-operative and six-month post-operative photograph of case 2 is shown in Figure 3 and Figure 4, respectively. There was no post-operative weakness in foot eversion, which was ascertained by muscle power assessment using the Medical Research Council (MRC) scale, with M5 power in all patients, assessed at eight weeks after surgery. Flap donor site healed well with a fine line scar. Skin graft donor site healed in all cases within three weeks. The phenomenon of auto-thinning of the muscle flap with time was seen in Figure 4.

## Discussion

Lower leg defects pose a challenge for reconstructive surgeons and usually require flap coverage due to the chance of tendon, bone, and implant exposure in this area, and the relative paucity of local tissue availability in this area. Available local fascio-cutaneous flaps often lead to bulky flaps with adjacent donor defects that can be unaesthetic [4]. For lower leg defects, the commonly used fascio-cutaneous flaps are distally based sural artery flap, peroneal propeller flap, reverse peroneal flap, posterior tibial propeller flap, etc, all of which have a donor area adjacent to the defect which needs to be skin grafted. Muscle flap options for lower leg are limited. Free flaps are commonly employed for lower leg defects, like the anterolateral thigh (ALT) flap, gracilis free flap, and medial sural artery perforator (MSAP) flap. ALT flap, being a fascio-cutaneous free flap is usually preferred when underlying secondary procedures, like bone grafting, are planned, but is bulky and needs multiple procedures for flap thinning. MSAP flap is a thin flap that contours well to the lower leg, but the donor defect sometimes needs skin grafting. Gracilis flap is another excellent choice of flap cover in lower leg and also contours well to the lower leg over time, due to auto-thinning due to muscle atrophy. Peroneus brevis muscle is an expendable muscle that can be a versatile option for small to moderate defects of lower leg. Being a muscle flap, it can resist infection and contour to the defect while causing minimum donor site morbidity. Loss of peroneus brevis, with a functional peroneus longus, doesn’t cause ankle instability [4,5]. Also, due to auto-thinning, these flaps can become less bulky over time leading to better aesthetic and functional outcomes. 

The peroneus brevis flap was first described as a proximally based flap by Pers and Medgyesi [6]. DBPBF was described by Mathes and Nahai [7]. Eren et al. published the first case series of DBPBF which had 19 cases from 1993-to 1999 [8], and the largest cases series to date is by Schmidt and Giessler (109 cases) from 1998 to 2009 [9]. Vascular anatomy of peroneus brevis is well studied and further modified by various cadaveric studies. It was initially described as type II muscle [1,10,11], but it was re-classified as type IV, and this vascular pattern was also proven by some authors, after conducting a series of cadaveric studies [2]. Angiographic studies in cadavers have shown that “as long as one of the pedicles is maintained, the complete filling of the muscle can be accomplished” [12].

Modifications of DBPBF like open book splitting method were shown to increase the flap coverage area to a maximum of 12 cm in length and 10 cm in width. In this method, the muscle was harvested along with periosteum of fibula and the outer surface of the muscle was split to facilitate coverage of larger defects [13]. Proximally based peroneus brevis flap and osteo-muscular composite peroneus brevis muscle flaps are described in literature.

In our institution, DBPBFs are used in selected cases of lower one-third leg small to moderate defects. A total of six patients were studied in our series, out of which flap was fully viable in five patients (80%). In one patient, there was a non-critical flap loss, which was debrided and negative pressure wound therapy was initiated and repeat grafting was done. Table 2 gives a comparison of our results with studies of Schmidt et al., Bajantri et al., and Thammanagowda et al. [9,14,15].

**Table 2:** Comparison of our results with similar studies

Author and year	Number of cases	Partial graft loss managed conservatively	Partial graft loss which needed repeat grafting	Flap tip loss which needed further procedures	Critical flap loss
Schmidt and Giessler, 2010 [9]	109	7.3%	14.7%	16.5%	12%
Bajantri et al., 2013 [14]	39	-	2.5%	6.25%	10.2%
Thammannagowda et al., 2014 [15]	32	-	12.5%	15%	6.25%
This study	6	16.6%	33.3%	16.6%	-

Split skin grafting over the muscle was done in the same sitting in all our cases, and we have seen that the partial skin graft loss was there in good majority of cases. Authors feel this is due to some post-operative oozing underneath the skin graft, as the usual tie-over pressure dressings were not applied for fear of flap compression. Hence an alternative way is to place the skin graft once the flap has settled well, as a planned second procedure.

On comparing with these studies, it was found that flap tip loss, when used for medial malleolar defects, is well described, possibly due to the greater arc of rotation required and the resulting stretch [14,15].

## Conclusions

The distally based peroneus brevis flap is a useful option for selected cases of small to medium-sized lower one-third leg defects, with acceptable complications and negligible donor site morbidity. It would be wise to select alternative options for medial malleolar defects since flap tip loss for such defects is well described.
